# Patterns of MiRNA Expression in Arctic Charr Development

**DOI:** 10.1371/journal.pone.0106084

**Published:** 2014-08-29

**Authors:** Kalina H. Kapralova, Sigrídur Rut Franzdóttir, Hákon Jónsson, Sigurður S. Snorrason, Zophonías O. Jónsson

**Affiliations:** Institute of Life- and Environmental Sciences, University of Iceland, Reykjavík, Iceland; CNRS UMR7622 & University Paris 6 Pierre-et-Marie-Curie, France

## Abstract

Micro-RNAs (miRNAs) are now recognized as a major class of developmental regulators. Sequences of many miRNAs are highly conserved, yet they often exhibit temporal and spatial heterogeneity in expression among species and have been proposed as an important reservoir for adaptive evolution and divergence. With this in mind we studied miRNA expression during embryonic development of offspring from two contrasting morphs of the highly polymorphic salmonid Arctic charr (*Salvelinus alpinus*), a small benthic morph from Lake Thingvallavatn (SB) and an aquaculture stock (AC). These morphs differ extensively in morphology and adult body size. We established offspring groups of the two morphs and sampled at several time points during development. Four time points (3 embryonic and one just before first feeding) were selected for high-throughput small-RNA sequencing. We identified a total of 326 conserved and 427 novel miRNA candidates in Arctic charr, of which 51 conserved and 6 novel miRNA candidates were differentially expressed among developmental stages. Furthermore, 53 known and 19 novel miRNAs showed significantly different levels of expression in the two contrasting morphs. Hierarchical clustering of the 53 conserved miRNAs revealed that the expression differences are confined to the embryonic stages, where miRNAs such as sal-miR-130, 30, 451, 133, 26 and 199a were highly expressed in AC, whereas sal-miR-146, 183, 206 and 196a were highly expressed in SB embryos. The majority of these miRNAs have previously been found to be involved in key developmental processes in other species such as development of brain and sensory epithelia, skeletogenesis and myogenesis. Four of the novel miRNA candidates were only detected in either AC or SB. miRNA candidates identified in this study will be combined with available mRNA expression data to identify potential targets and involvement in developmental regulation.

## Introduction

Since the initial discoveries of lin-4 and let-7 miRNAs have emerged as key regulators of animal development (reviewed in [1], [2]). These small (∼22 nt) non coding RNAs, regulate gene expression by inducing mRNA degradation or translational repression, making for a specific and “fine-tunable” response (reviewed in [3]). miRNAs originate from different parts of the genome (intergenic regions, exons or intronic sequences [4] and are transcribed either as independent transcriptional units or as clusters of several miRNAs (reviewed in [5]). A common feature of all miRNA genes, regardless of their genome location, is the folding of their primary transcript into a stem-loop structure. This hairpin structure is recognized and converted into a miRNA-miRNA* duplex by the miRNA processing machinery (see [5]). One of the strands, dubbed the “mature” miRNA, is then loaded into the miRISC complex while the complementary “star” sequence is often degraded [6]. In most cases of miRNA mediated gene regulation the target repertoire is determined by the “seed” region (nt 2-8 located at the 5' end of the mature miRNA) of the miRNA [7].

In general miRNAs are highly conserved among taxa [5]. Comparative studies show how new miRNAs have continuously been emerging during the evolution of metazoan genomes [8], [9] through various mechanisms including gene duplications of preexisting miRNAs followed by changes in their sequences, or *de novo* appearance from random hairpins [10]. However, once they become integrated into the regulatory network, their primary sequence and particularly their seed region, becomes subject to strict selective constraints [5], [11]. Variation in timing and expression patterns among species suggests that these molecules may play an important role in shaping physiological differences. For example comparison of two fish species (medaka and zebrafish) showed that heterochrony in miRNAs expression is associated with neuromast and craniofacial development [12]. This was suggested to reflect the differences in formation of the head and sensory epithelia observed between medaka and zebrafish. Morphological differences arising in development can potentially drive evolutionary change, adaptive divergence and speciation (discussed in [1]). More specifically, it has been suggested that miRNAs may generally cover more restricted regulatory niches than transcription factors and thus frequently be more important in terminal differentiation programs [13]. It has also been proposed that miRNAs are involved in enhancing species evolvability by stabilizing gene expression and signaling cascades leading to the increased distinctness of developmental phenotypes, thereby increasing heritability of traits and facilitating natural selection ([14] and discussion in [15]).

### Arctic charr as a model species to study adaptive divergence

The high level of phenotypic polymorphism present in Northern freshwater systems offers an excellent opportunity to study adaptive divergence [16]. These watersheds, with their rivers and lakes, were formed after the last glacial epoch 10 000–16 000 years ago. The short evolutionary history characterized by physical variability and topographic dynamics sets a stage where the early steps of divergence may be playing out in multiple locations and species. Studies of whitefish (*Coregonus clupeaformis*), threespine stickleback (*Gasterostreus aculeatus*) and Arctic charr (*Salivelunus alpinus*) have shown that fish inhabiting these systems exhibit an extremely high level of inter-population variation in phenotype with many populations diversifying along a benthic to limnetic habitat axis [17]–[20]. Although Arctic charr in Iceland originates from a single Atlantic lineage [21], this species shows an extremely high level of variation in phenotype between populations and many examples of polymorphism (i.e. sympatric morphs) have been documented [17], [22]–[24]. The Arctic charr morphs of Lake Thingvallavatn constitute an extreme example of local phenotypic diversity. Four morphs grouped into two morphotypes have been described in the lake: a limnetic morphotype represented by planktivorous (PL) and piscivorous (PI) charr, with pointed snout and evenly protruding jaws, and a derived, benthic morphotype represented by small (SB) and large benthivorous (LB) charr, blunt snout, short lower jaw and relatively large pectoral fins [25]. These morphs also differ extensively in life history characteristics (size and age at maturity) and embryology [26]–[29]. The morphs also exhibit strikingly clear differentiation in ecology as reflected in different habitat use, diet and endoparasite fauna [27], [28], [30]. Several common garden experiments have shown that some key morph specific traits have a definite genetic basis [31], [32]. A recent study, using neutral microsatellite markers, revealed significant but subtle genetic differentiation between the three most common morphs in Lake Thingvallavatn, which is consistent with a scenario of early evolution of reproductive isolation, followed by slow divergence by drift with restrictive gene flow [33]. Notably, a study of immune system genes revealed more pronounced genetic differentiation among the morphs in the lake, consistent with a scenario where parts of the immune systems have diverged substantially among Arctic charr morphs from Lake Thingvallavatn [34]. The adaptive nature of the trophic morphology and feeding behavior of the Thingvallavatn morphs has been demonstrated in a series of laboratory rearing experiments [29], [31], [35]. Moreover the role of developmental heterochrony in the evolution of the Thingvallavatn Arctic charr morphs was demonstrated in a study showing that some skeletal elements of the head start ossifying earlier and/or faster in small benthivorous embryos than in embryos derived from the planktivorous morph [35].

**Figure 1:**
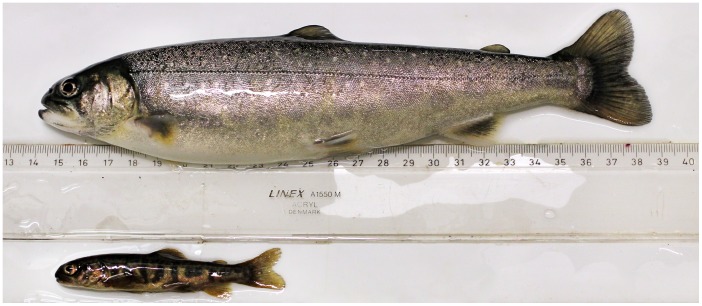
Two contrasting Arctic charr morphs differing in size, coloration and head morphology. Top: Arctic charr from aquaculture stock (AC) is large, silvery and has a pointed snout and long lower jaw, Bottom: Small benthic charr from Thingvallavatn (SB) is small, dark and has a sub-terminal mouth and rounded snout.

Some of the key differences in functional traits that define the charr morphs are without doubt rooted in differences in the expression of developmental genes. We hypothesize that miRNAs may, through their potentially stabilizing effect of phenotypes [14], play a fundamental role in the divergence of developmental processes that induce differential cranial morphologies in Arctic charr morphs. As a first step of addressing this hypothesis we utilized high-throughput sequencing techniques to identify and annotate Arctic charr miRNAs and to study their expression during the development of two contrasting Arctic charr morphologies. To this end we used a common garden set up to generate embryonic series of two contrasting Arctic charr morphotypes, a benthic morphotype, represented by the SB-charr from Thingvallavatn and a limnetic morphotype represented by fish from the Hólar aquaculture stock (AC). These two morphs differ greatly in adult size, color and head morphology (Figure 1): SB are small, dark and have a sub-terminal mouth and rounded snout whereas AC are large, silvery and have a pointed snout and a longer lower jaw. We sampled AC and SB embryos at four developmental time-points reflecting important events in Arctic charr craniofacial development and used high-throughput sequencing to quantify differences in miRNA expression between the morphs. More specifically we identified and annotated Arctic charr miRNAs using homology to known miRNAs in other species. Furthermore, we identified a large set of novel miRNA candidates by aligning reads to genomic sequences from the closely related Atlantic salmon, *Salmo salar.* Expression levels for both known and novel miRNAs were compared between AC and SB.

## Materials and Methods

### Sampling and methodology

All sampling from the wild and rearing in aquaculture was performed according to Icelandic law and with proper permissions. Fish from Lake Thingvallavatn were caught by the authors for the purpose of this study with fishing permissions obtained from the Thingvellir National Park Commission and the owner of the Mjóanes farm. SSS and ZOJ hold special permits for sampling fish from nature for scientific purposes according to Icelandic law (clause 26 of law 61/2006 on salmonid fishing). Control fish from Hólar aquaculture stock were obtained from a national breeding programme, and were not specifically bred for the purpose of this project. These fish are held at the arctic charr breeding station, a quarantined rearing and holding facility, at Hólar University College. After stripping for gametes, parent fish were killed by a sharp blow to the head and checked for absence of breathing when placed in water. Setting up crosses and the subsequent killing of parents was performed by the authors. Ethics committee approval is not needed for regular or scientific fishing in Iceland (The Icelandic law on animal protection, Law 15/1994, last updated with Law 157/2012). The rearing of embryos was performed according to Icelandic regulations (licence granted to Hólar University College aquaculture and experimental facilities) in Verið, Sauðárkrókur, Iceland. Sampling of embryos was performed by University College Aquaculture Research Station (HUC-ARC) personnel. HUC-ARC has an operational license according to Icelandic law on aquaculture (Law 71/2008), that includes clauses of best practices for animal care and experiments. For this study the last gestation age at which embryos were sacrificed was 434 (τ_s_) units. For RNA extraction, samples were flash frozen in RNA later. Prior to freezing eggs were permeabilized by puncture with a needle. Samples for staining (not described in this study) were treated with an overdose of phenoxyethoanol before fixing.

For this study we used developmental time-series from pure crosses of two Arctic charr morphs, Hólar aquaculture charr (AC) and small benthic charr (SB) from Lake Thingvallavatn. These strains were selected mainly for their pronounced differences in body size, coloration and head morphology (Figure 1). As stated above, the AC crosses were made with parents from the Hólar breeding programme [36]. Fish from the small benthic morph (SB) were caught in Lake Thingvallavatn using gill-nets. Eggs from several females were pooled and fertilized using milt from several males from the same group. Eggs were reared at approximately 5°C in a hatching tray (EWOS, Norway) under constant water flow and in complete darkness at the Holar University College experimental facilities in Verið, Sauðárkrókur. Exact water temperature was recorded twice daily to estimate the relative age of the embryos using tau-somite (τ_s_) units defined as the time it takes for one somite pair to form at a given temperature [37]. Embryos were collected throughout development and either fixed in 4% PFA or stored in RNAlater (Ambion) at −80°C. Based on embryos sampled at different developmental stages and stained with alcian blue (cartilage) and alizarin red (bone), four time-points (141, 161, 200 and 434 τ_s_) were selected to represent important stages of bone and cartilage development. Stages 141, 161 and 200 are embryonic whereas stage 434 is a fry stage and for simplicity these stages will be referred to as stages 1, 2, 3 and 4, respectively. Two independent samplings were performed: one was used for high-throughput small-RNA-sequencing (miRNA-seq) and the other one for qRT-PCR.

### Small RNA sequencing

Total RNA from each stage of each morph was isolated from a pool of 6 whole embryos and enriched for small RNAs using the mirVana kit (Ambion). The purity and amount of small RNA was verified on a BioAnalyzer (Agilent Technologies). The samples were prepared for sequencing following the small RNA v1.5 sample preparation protocol from Illumina. Briefly, 3‘ and 5‘ RNA adapters were ligated to small RNAs, which were subsequently, reverse transcribed into DNA and PCR amplified. The samples were then run on polyacrylamide gels and the DNA eluted from bands corresponding to 20–30 nucleotide RNA fragments. miRNA and transcriptome sequencing (mRNA-seq) was performed at deCODE Genetics (Reykjavik, Iceland) using the TruSeq smallRNA (v1.5) kit (Illumina) on an Illumina GAII_X_ instrument. Raw reads were submitted to NCBI Sequence Read Archive (SRA) under accession number SRP039492.

### miRNA-seq data processing

Raw reads were processed with cutadapt [38] as follows: First, adaptor sequences were removed and only reads with adaptors were kept. Next, we used the FastX toolkit (http://hannonlab.cshl.edu/fastx_toolkit/index.html) (script available on request) and the quality scores associated with the reads to remove bases with a Phred based quality score [39] of less than 20 from read ends. Sequences retaining less than 15 nucleotides after filtering were discarded. Reads where 10% or more of the bases had a Phred quality score lower than 20 were also discarded. Finally, identical reads were reduced to one copy with the redundancy noted in the read name. The sequence filtering and collapsing was repeated for each sample.

### Annotation of ncRNAs

To annotate sequences using known RNAs we used Rfam version 10.1 [40] and miRBase [41]–[44] version 20 databases. The Rfam database was searched with HMMER (version 3.0; http://hmmer.janelia.org/) with an e-value cutoff of 0.01. For the miRBase the ssearch command from the fasta package version 36.3.6d [45] was used to detect homology between the mature miRNAs and the collapsed sequences (e-value cutoff 0.01).

### On a quest for novel miRNAs

To identify novel miRNAs we used a probabilistic model of miRNA biosynthesis implemented in miRDeep 2 [46]. As a sequenced Arctic charr genome is not currently available, we used the genome sequences from the closely related Atlantic salmon [47] for reference. Collapsed reads were aligned to the Atlantic salmon genome with the mirDeep 2 mapping program (10 minimum reads per miRNA) for both morphs with the time-points combined. To facilitate mapping, collapsed sequences with strictly lower read count than 4 were omitted from the detection. The miRDeep2 algorithm then uses the reference regions bracketing the aligned reads to compute a hairpin structure and estimates the probability of each sequence being a true miRNA precursor based on the position of the reads, their frequency, the energetic stability of their secondary structure and conservation of the 5′ ends.

Sequences with log score greater or equal to 2 were considered as potential miRNAs, the predicted hairpins were searched against hairpins from mirBase (version 20) with blastall (version 2.2.26, -W 7) [48]. Candidates were annotated as known miRNAs if the alignment length was greater or equal to 60 nucleotides and expected value for the match was lower than 0.01 (-e 0.01) otherwise the hairpins were classified as novel.

### PCR amplification and sequencing of miRNA clusters

To assess the degree of sequence conservation for genomic clusters containing known and novel miRNAs between Arctic charr and Atlantic salmon we selected 4 clusters containing known miRNAs (miR-19c, 18b* and 20b; miR-133a and miR-133b and miR-143-3p and miR-143-5p; miR-219-3p and miR-219-5p) and 3 clusters containing novel miRNA candidates (sal-nov-235, sal-nov-242 and sal-nov-343) and PCR amplified their genomic regions from the Arctic charr genome. Primers were designed with Primer3 (http://primer3.wi.mit.edu/) (Table S1). The same PCR program was used for all primer pairs: an initial denaturation at 95°C for 5 min; 35 cycles of 95°C for 45 seconds; 45 seconds at a 53°C; 1 min at 72°C, then a final step of 10 min at 72°C. PCR products were treated with ExoSap and sequenced on an Applied Biosystems 3500xL Genetic Analyzer using BigDye chemistry. Raw sequencing data was base-called by Sequencing Analysis Software v5.4 with KBTMBasecaller v1.41 (Applied Biosystems), and run through Phred and Phrap, prior to trimming primer sequences, visual editing of ambiguous bases and putative polymorphisms in Consed [49]. Fasta files were exported and aligned with ClustalW (http://www.ebi.ac.uk/Tools/msa/clustalw2/, and manually inspected for alignment errors in Genedoc (www.psc.edu/biomed/genedoc). All sequences were deposited in Genebank under the accession numbers [KJ573796-KJ573802]. These sequences were then searched using blast against the salmon database. The conservation between Arctic charr and salmon ranged between 91–94% for the known miRNAs and 92–98% for the novel miRNA candidates. Mismatches were always located outside of the miRNA mature-star sequence.

### Differential expression analysis

The R package edgeR [50] was used to study the differential expression of conserved and novel miRNA candidates between morphs and among developmental time-points in a generalized linear model, where the additive covariates (no-interaction) corresponded to developmental time-point and different morphs. The normalization factors were calculated for each sample using the function calcNormFactors. As there are no replicates in any of the experimental conditions, the options method = “deviance”, robust = TRUE and subset = NULL were used for estimating the common dispersion (function estimateGLMCommonDisp) parameters as recommended by the edgeR user manual. The trended and tagwise dispersion were also estimated (function estimateGLMTrendedDisp and estimateGLMTagwiseDisp) with default options. The statistical significance of the terms was assessed by comparing likelihood difference to a reduced model without the time or the morph terms, with the function glmLRT in edgeR. The first 20 bases of each annotated sequence (novel and previously described miRNAs) were used as an identifier and the counts were aggregated for sequences that share the first 20 bases. This allowed us to work at the sequence level without lumping together isoforms (isomiRs). An entry (first 20 bases) was only considered for the statistical testing if the counts per million reads were strictly greater than 3 in at least two experimental points resulting in 1862 tags. We adjusted for multiple testing using the Benjamini-Hochberg false discovery rate [51]. The R script used for this analysis is available in File S1.

### Descriptive analysis

Cluster analysis was performed using the heatmap function and plotted using the gplots package in R (http://www.r-project.org/). Prior to the clustering analysis expression levels for each miRNA were normalized across samples using a Variance Stabilizing Transformation.

### Real-time quantitative PCR analysis

In order to verify the observed differential expression between morphs in our miRNA-seq data, we selected 9 miRNAs (sal-miR-17, sal-miR-26a, sal-miR-30b, sal-miR-122, sal-miR-140, sal-miR-181a*, sal-miR-196a, sal-miR-199a and sal-miR-206) for qPCR analysis. We concentrated on the 3 embryonic stages, as in both our analyses (for morph or developmental effect) the expression profiles between the samples of the last stage appeared to be very similar (see results). For the qPCR analysis two separate RNA extractions (biological replicates) were used for each data point. RNA was extracted from pools of 6 whole embryos/fry using a standard TRI Reagent (Sigma) protocol and treated with DNaseI (New England Biolabs) in order to limit genomic DNA contamination. All samples were from the same sampling effort and were extracted and processed simultaneously. cDNA was synthesized using the Exiqon universal cDNA Synthesis Kit II. The consistency of the cDNA synthesis among samples was verified using a spike in template along with a Control primer set (Exiqon). For the qPCRs we used SYBR Green master mix (Exiqon) and LNA primers (Exiqon). All qPCRs were done in duplicates (technical replicates) in a 10 µl reaction volume in 96 well-PCR plates on an ABI 7500 real-time PCR System (Applied Biosystems) following manufacturer instructions (Exiqon). The same PCR program was used for all miRNA primer pairs: starting with a 2 min hold at 50°C followed by a 10 min initial denaturation at 95°C and 45 cycles of 10 sec denaturation at 95°C and 1 min annealing/extension at 60°C. A melting curve analysis was performed at the end of each PCR to verify the specificity of the amplification. U2 spliceosomal snRNA (Primer sequence: GGTACTGCAATACCGGGG) was initially selected as a reference gene. However, the use of non-miRNA genes as reference has been shown to be problematic and the use of mean expression is often more appropriate [52]. We therefore opted to use the geometric mean for the expression values of the miRNAs under study as a reference. Relative expression (fold change) for each miRNA compared to stage 1 in AC was calculated in R using a script provided in File S2.

## Results

### Small RNA sequencing descriptive statistics

In order to identify miRNAs involved in Arctic charr development and morph differences, we made 8 small RNA libraries from four developmental time-points of two contrasting morphs of Arctic charr. The sequencing depth ranged from 29.1 to 33.9 million reads with a mean depth of 32.4 million reads per sample. After removing the adapters using cutadapt [38] and filtering out low quality reads using the FastX toolkit (http://hannonlab.cshl.edu/fastx_toolkit/), we obtained on average 24.8 million reads per sample (Table 1). The size distribution of the collapsed reads of all 8 libraries accounting for redundancy is shown in Figure S1. The majority of the reads were 21–23 nucleotides, corresponding to the typical miRNA size range. Details of the size distribution for both unique and collapsed reads for all 8 libraries are shown in Figure S2. All 8 libraries showed similar distribution with a peak at 21–23 nt. Furthermore, annotation of the collapsed reads using the Rfam database confirmed that our small RNA libraries were highly enriched with miRNAs (Table 2).

**Table 1:** Summary of read numbers from small RNA sequencing.

Sample	Number of reads (NR)	NR after adapter trimming	NR after collapsing
SB 1	33.4 M	23.1 M	2.4 M
SB 2	32.8 M	28.2 M	0.9 M
SB 3	33.9 M	30.5 M	0.7 M
SB 4	29.1 M	21.6 M	0.7 M
AC 1	32.8 M	24.2 M	1.7 M
AC 2	32.5 M	21.1 M	2.0 M
AC 3	31.3 M	23.2 M	0.7 M
AC 4	33.6 M	26.2 M	1.0 M

Number of reads (NR, in millions of reads) in high-throughput data from small RNA libraries of four developmental points and two morphs of Arctic charr. AC  =  Aquaculture charr, SB  =  Small Benthic charr.

**Table 2:** High-throughput reads annotated using the Rfam database.

snc RNA	Number of reads
miRNA	50841311
rRNA	49033
mRNA	5755
tRNA	24451
SNORD	151004
U	208259
sno	25857

### A total of 326 conserved and 427 novel miRNA candidates were found in the data

**Figure 2:**
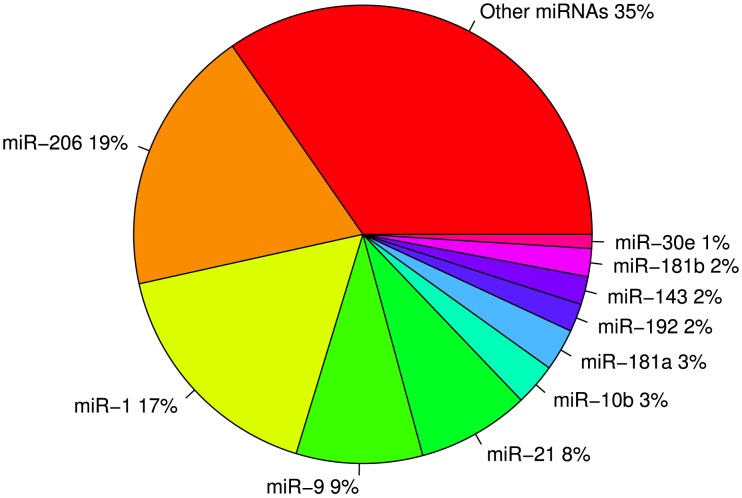
Relative abundance of known miRNAs in all samples combined. Together the 10 most abundant known miRNAs constitute 65% of all known miRNAs.

All collapsed reads were compared to the mature miRNA sequences available in miRBase (release 19) using ssearch [45] and 326 candidates (Table S2) were identified with high confidence (e-value <0.001). The 10 most abundant miRNAs account for 65% of the total conserved miRNAs (Figure 2) with sal-miR-206 and sal-miR-1 alone accounting for 36% of the total miRNAs. We identified 427 novel miRNA candidates (Table S3) of which 37% were represented by the 10 highest expressed putative miRNAs. We sequenced the genomic regions of three novel miRNAs (sal-nov-235, sal-nov-242 and sal-nov-334). They were all highly conserved between Arctic charr and Salmon (Table S1). Furthermore their mature and star sequences are located in highly conserved blocks in medaka, fugu, tetraodon and stickleback. Several of the conserved miRNAs were present in two or three isoforms (isomiRs). For example sal-miR-451 exists in 3 isoforms (Table S2). Two of these (sal-miR-451_1 and sal-miR-451_3) are highly conserved among vertebrates, whereas the third (sal-miR-451_2) has not previously been described in other species. This derived isoform differs in one base (G->U substitution) located at the 3' end of the mature sequence and is the predominant isoform of sal-miR-451 in our data (Table S2). Another interesting example is sal-miR-152, where 4 isoforms are found in our data (Table S2) with the most abundant being the ancestor sequence. The three other isoforms are one mutation away from the ancestral form. Interestingly, these mutations (T->A, C, or G) are located at the same site (position 5) for all 3 derived isoforms (Table S2).

### 51 known miRNAs and 6 novel miRNA candidates are differentially expressed among developmental stages

**Figure 3:**
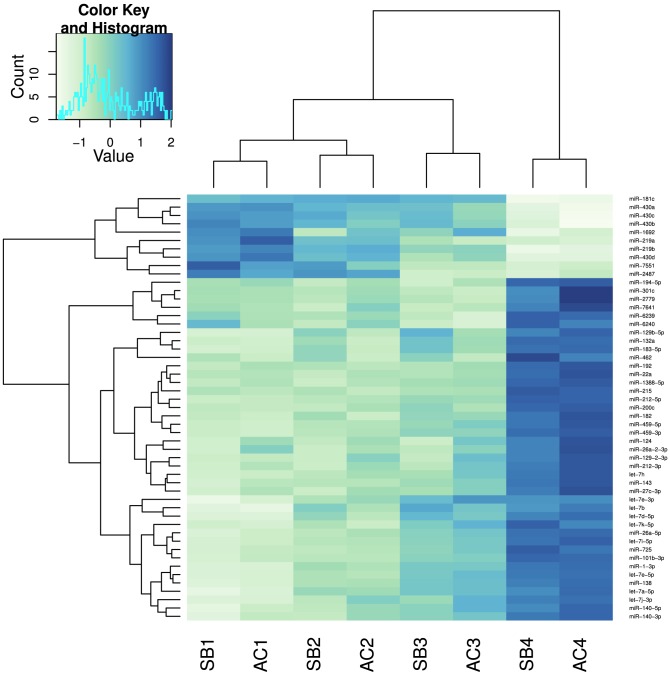
Heat-map showing relative expression of the 51 miRNAs significantly differentially expressed among developmental stages. Expression levels for each miRNA were normalized across samples using variance stabilizing transformation. Blue denotes high and white low relative expression. AC stands for Aquaculture charr and SB stands for Small benthic charr. Numbers 1, 2, 3 and 4 depict the four developmental time-points.

We found 51 known miRNAs to be differentially expressed among developmental time-points. Hierarchical clustering (Figure 3, see also Table S4 for background data) of miRNA expression showed that the 8 samples grouped into four clusters according to developmental time: one major division separates the three embryonic stages (1, 2 and 3) from the last post-hatching stage (4) and three divisions for each of the three embryonic stages (Figure 3). This major division between embryonic and post hatching stages indicates a clear shift in miRNAs expression between these developmental phases. The second division separates stage 3 from stages 1 and 2 and the third division separates stages 1 and 2 (Figure 3). Interestingly, there are two major divisions in the miRNA expression pattern clustering: node one depicts miRNAs that are highly expressed during the embryonic stages and their expression decreases in the last stage while the second node includes miRNAs with high expression in stage 4 and low expression in the embryonic stages. For example members of the 430 family (miR-430 a, b, c and d) are highly expressed in the embryonic stages and their expression decreases markedly in late development. In addition other miRNAs, such as sal-miR-219 a and b and miR-181c, show higher expression in the embryonic stages. On the other hand, miRNAs such as sal-miR-22a, 140, 182, 183, 192, 215 and different members of the let-7 family show increasing expression over time. Of the novel candidates, 6 putative miRNAs were found to be differentially expressed among developmental points (Table 3). Three of them sal-nov-1, sal-nov-5 and sal-nov-18 are also differentially expressed between morphs.

**Table:** **Table 3.** Novel miRNA candidates differentially represented between morphs and/or developmental stages in the small-RNA-seq data.

Name	Expressed	SB1	SB2	SB3	SB4	AC1	AC2	AC3	AC4
sal-nov_1	Morph/Time	7	4	1	1	17	31	25	10
sal-nov_2	Morph	8	13	16	9	104	89	151	88
sal-nov_3	Morph	34	78	63	17	0	0	0	0
sal-nov_4	Morph	0	0	0	0	13076	5397	5755	560
sal-nov_5	Morph/Time	0	0	0	0	1800	563	343	0
sal-nov_6	Morph	47	9	14	18	73	58	23	28
sal-nov_7	Morph	25	5	1	9	48	45	14	5
sal-nov_8	Morph	207	168	20	7	256	117	47	71
sal-nov_9	Morph	130	59	13	54	268	361	173	70
sal-nov_10	Morph	16	6	12	10	45	53	52	25
sal-nov_11	Morph	8	13	16	9	104	89	151	88
sal-nov_12	Morph	8	12	13	6	14	17	15	6
sal-nov_13	Morph	21	44	112	133	113	110	236	308
sal-nov_14	Morph	2	32	31	53	27	25	96	103
sal-nov_15	Morph	3	22	32	31	6	7	8	10
sal-nov_16	Morph	644	1472	1725	983	966	648	944	1011
sal-nov_17	Morph	24	30	27	2	65	41	69	50
sal-nov_18	Morph/Time	13	89	121	372	3	11	17	219
sal-nov_19	Morph	8	4	9	0	207	63	107	15
sal-nov_20	Time	70	274	967	5358	128	213	988	6078
sal-nov_21	Time	3472	7090	12625	26369	5005	4127	10188	20766
sal-nov_22	Time	121	107	56	1	206	71	26	0

Included are miRNA names, differential expression and number of raw reads per stage per morph.

### 53 known miRNAs and 19 novel miRNA candidates are differentially expressed between AC and SB embryos

**Figure 4:**
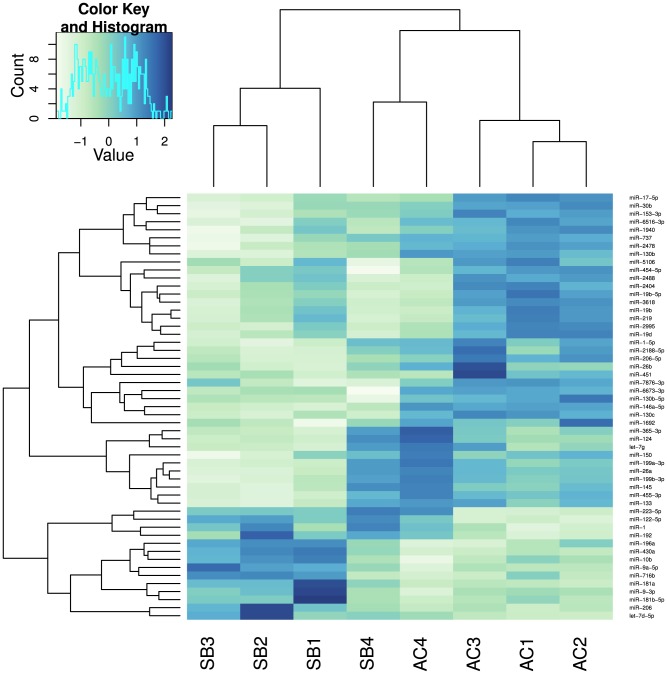
Heat-map showing relative expression of the 53 miRNAs significantly differentially expressed between AC and SB morphs. Expression levels for each miRNA were normalized across samples using variance stabilizing transformation. Blue denotes high and white low relative expression. AC stands for Aquaculture charr and SB stands for Small benthic charr. Numbers 1, 2, 3 and 4 depict the four developmental points.

**Figure 5:**
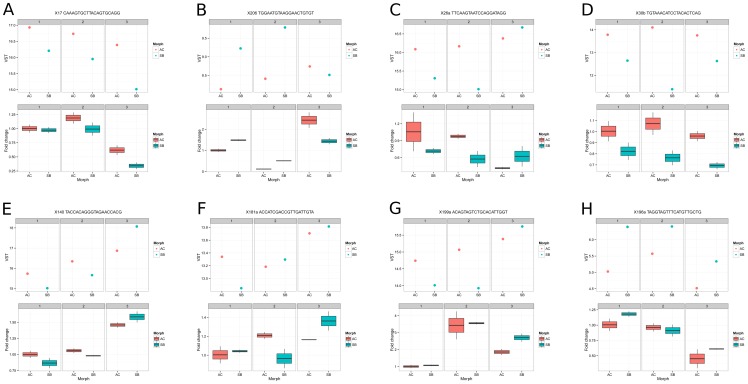
Comparison of expression of 8 selected miRNAs (miR-17, 26a, 30b, 140, 181a, 196a and 199a) at three developmental time-points for two contrasting Arctic charr morphs (AC and SB) quantified by small RNA-seq (upper panel) or qPCR (lower panel).

We tested for differential expression between morphs using a Generalised Linear Model and adjusted for multiple testing using the Benjamin-Hochberg false discovery rate as decribed in methods. We found 53 miRNAs to be differentially expressed between AC and SB. These miRNAs cluster by morph during the embryonic stages (stages 1-3) (Figure 4, see also Table S4 for background data). During these 3 stages miRNAs such as sal-miR-130, 133, 153, 17, 30, 451, 219, 26, 199a and 145 are highly expressed in AC, whereas sal-miR- 206, 133, 122, 181a, 192, 196a and 223 are highly expressed in SB. The expression of some of these “morph specific” miRNAs for example sal-miR-130, 153, 17, 30b and 30c in AC and sal-miR-196a, 206, 192 and 122 in SB observed in the embryonic stages decreases markedly in the last stage. During the last stage the observed miRNA expression differences between the two morphs disappear (Figure 4). Of the novel miRNA candidates, 19 putative miRNAs were found to be differentially expressed between AC and SB (Table 3). Two of them, sal-nov-4 and 5 were only expressed in AC and at most/all stages whereas expression of another putative novel miRNA, sal-nov-3, was only detected in SB offspring and at all four developmental points. With three exceptions (sal-nov-4, 5 and 16) all of the differentially expressed putative miRNAs showed very low expression levels (Table 3). We selected 9 miRNAs and further studied their expression by qPCR using independent biological replicates. The selection was based on the dynamics and degree of differential expression between morphs and/or among developmental points seen in the sequencing data. We concentrated on the three embryonic stages, as in both our analyses (for morph or developmental effect), the expression profiles between the samples of stage as the expression profiles between the samples of stage 4 appeared to be very similar. Eight of these miRNAs (miR-17, miR-26a, miR-30b, miR-140, miR-181a*, miR-196a, miR-199a and miR-206) amplified well (Figure 5), whereas miR-122 showed double peaks in melting curve analysis and was discarded from further analysis. Five (miR 17, 26a, 30b, 140 and 206) out of eight miRNAs tested with qPCR showed similar expression patterns to what was expected from the high-throughput sequencing (Figure 5, A–E). Three miRNAs (miR-196a and miR-199a and miR-181a) exhibited similar expression patterns in one or two of the three stages under study, (Figure 5, F–H).

## Discussion

The molecular mechanisms underlying the development of Arctic charr morphologies are likely to have parallels in other vertebrate species and studying them is of interest in both developmental and evolutionary contexts. Given the recent divergence in northern populations of Arctic charr, it is likely that the observed phenotypic polymorphism rooted in development in this species arose mostly by differences in gene regulation as opposed to changes in protein coding sequences. In a recent study [53] two genes involved in matrix remodeling in bone formation (sparc and mmp2) showed consistent differences in expression during the development of embryos derived from benthic and limnetic morphs of Arctic charr, suggesting that these genes might be involved in the development of these distinct Arctic charr morphologies. Little is known about what controls such differences in expression. While numerous studies demonstrate the involvement of transcription factors and other regulatory elements in the phenotypic evolution of birds [54] and fish [55]–[60], there are few known examples of miRNAs playing a role in morphological variation. In a recent study Arif *et al.* [61] experimentally demonstrated that differences in the “naked valley” phenotype observed among natural populations of *D. melanogaster* were caused by variation of miR-92a expression. Our study is the first phase of assessing the involvement of miRNAs in the development of key morphological traits and their potential role in the morphological evolution of the highly polymorphic Arctic charr. In so doing we also hope to shed light on some of the developmental circuitry operating at these levels of development.

Using small RNA-seq we found 326 known and 427 novel miRNA candidates in Arctic charr. A few of the candidates, termed novel (Tables 3 and S2) are absent from miRbase but have been previously identified in other salmonid species [62]–[64]. When only the 326 known miRNAs are considered, the 10 most abundant ones account for 65% of reads (Figure 2). These miRNA are highly conserved among taxa and have been shown to have important functions during development. The two most abundant miRNAs in our data, miR-206 and miR-1, together account for 36% of the total known miRNAs (Figure 2). Their role in skeletogenesis and myogenesis has been studied in some detail, for example miR-206 has been found to induce myogenic differentiation [65]–[67] while inhibiting osteoblast differentiation [68], and miR-1 has been found to regulate skeletal muscle and cardiac development [69], [70]. These miRNAs are highly conserved in animal evolution [8] with miR-1 retaining its muscle-specific expression from *C. elegans* to human [71]. Other highly expressed miRNAs are involved in cardiogenesis (miR-21), neurogenesis (miR-9), gut and gall bladder development (miR-143 and miR-192) [72]. One of the oldest miRNAs in the animal kingdom (miR-10) [8] is also among the 10 most highly expressed miRNAs in our data. Encoded in the intron of hoxB4, this miRNA is suggested to play a role in anterior posterior patterning.

A few miRNAs exist in multiple forms in our data. Among these the most interesting examples are miR-152 and miR-451. In the case of miR-152 the polymorphism is located in the seed region, which suggests functional divergence. In the case of miR-451 the ancestral and derived forms differ in one base (G->U substitution) located in the 3' end of the mature sequence. Interestingly the derived form of miR-451 is also the most abundant one. Although not as essential as the seed region, 3' miRNA-target pairing has a role in defining target specificity within miRNA families [73]. The derived form of miR-451 might have evolved following the whole genome duplication Salmonids underwent 25–100 million years ago or as a result of gene duplication. Other possibilities for the presence of this miRNA in our data include post-transcriptional editing of the ancestral form. However, without a sequenced Arctic charr genome, distinguishing between evolutionary scenarios represents a challenge. The derived form of mir-451 might be specific to Arctic charr as it is not present in the salmon genome and has not been reported in rainbow trout. As miRNAs often co-evolve with their targets [5] further phylogenetic analysis will help shedding light on the evolution of miR-152 and miR-451 and their targets in Arctic charr.

51 previously annotated miRNAs were found to be differentially expressed among developmental points. The cluster analysis of these miRNAs showed a clear shift in miRNA expression between the embryonic stages and the post-hatching stage, visible from both dendrograms. Although samples grouped by developmental stage, the major division was between stages 1, 2, 3 and stage 4. These findings were further confirmed by the existence of two major clades of miRNA expression: one containing miRNAs highly expressed during the embryonic stages and one the last stage. Among the miRNAs showing high expression in early development are the members of the 430 family (miR-430 a, b, c and d). In zebrafish these miRNAs are involved in the maternal to zygotic transition by deadenylation and clearing of maternal transcripts [74]. The majority of the miRNAs showed higher expression in the last developmental stage. Examples include miR-1, members of the let-7 family, miR-22a, miR-140, miR-182, miR-183, miR-192 and miR-215. The evolutionarily ancient and highly conserved let-7 family is involved in the regulation of the timing of developmental events in *C. elegans*, in particular the transition from larval stage 4 (L4) to adult [75]. In vertebrate development let-7 is temporally regulated and it is thought to play a role in late temporal transitions during development [75]. Other miRNAs found to be highly expressed in the last developmental stage are also involved in major developmental processes such as muscle differentiation (miR-1), endochondrial bone development (miR-140) and neuromast differentiation (miR-182 and 183) [12], [82]. Overall 72 miRNAs (19 novel miRNA candidates and 53 conserved miRNAs) were found to be differentially expressed between AC and SB at the developmental points under study. Of those sal-miR-196a, sal-miR-206, 122, 192, 196a, 223 and 181a were more highly expressed in SB whereas sal-miR-26a, 30b, 17-5p, 153-3p, 130b and c, 199a were more highly expressed in AC (Figure 4). All of the conserved miRNAs showing variation in expression between the Arctic charr morphs have been found to play an important role in development. For example, miR-196a, which is encoded in a Hox cluster, has been found to be involved in axial and appendicular patterning in chicken [76] and zebrafish [77]. Another muscle specific miRNA, miR-206, shows large expression differences between SB and AC especially at stage 3, where there is a 2.5 fold difference between the two morphs. This miRNA is involved in muscle differentiation and its expression is up-regulated by MyoD in differentiating muscle fibers. Loss of function of MyoD leads to down-regulation of miR-206 and severe deformities in the craniofacial elements [78]. miR-206 has also been shown to directly affect osteoblast differentiation and its overexpression in the osteoblasts of transgenic mice leads to bone abnormalities [68].

Other conserved miRNAs, miR-130b and c, miR-133, miR-199a-3p, miR-26a and miR-451 were highly expressed in AC throughout early development compared to SB. Some of these miRNAs are involved in myogenesis and skeletogenesis, for example, miR-199a-3p is important for normal skeletal development. In mouse a knockdown of Dnm3os (the primary precursor of a miR-214-miR-199a cluster) leads to skeletal abnormalities such as craniofacial hypoplasia [79]. miR-26 contributes to neurogenesis and myogenesis [80] and is involved in rainbow trout embryonic development [62] whereas miR-451 has been found to be involved in erythroid maturation in zebrafish [81]. Nineteen novel miRNA candidates were found to be differentially expressed between the two Arctic charr morphs in this study. Of those, two were only expressed in AC while one putative miRNA was only expressed in SB. These novel miRNA candidates were detected in most/all stages in one morph and not detectable in any of the stages in the other morph (Table 3), therefore it is unlikely that they represent a sequencing or technical error. As we used the Salmon genome to detect novel miRNAs, none of the novel miRNAs is likely to be morph or even Arctic charr specific, although expression differences can be expected. Several scenarios exist as to why these miRNAs are not expressed in both morphs, for example they might have been lost, their sequence might have been modified leading to the instability of the miRNA secondary structure or their expression repressed.

miRNAs are understudied in fishes and at present represent just a fraction of the miRNAs described in mammals. In the latest release of mirBase (version 20) from June 2013 there are only 255 mature miRNA sequences available for zebrafish, whereas 2578 mature sequences have been described in humans. Here we find 427 novel miRNA candidates, which are not Arctic charr specific. Some of these miRNAs are in highly conserved blocks (sal-nov235, 242, 334) among fishes, indicating that these miRNAs might have arisen early in the evolutionary history of fishes.

## Concluding Remarks and Future Directions

The theoretical underpinnings of our study are based on the general proposition that differences in the level, timing and pattern of miRNA expression or acquisition of new miRNAs can influence variation in developmental circuits, so as to generate diverse and possibly discrete morphological phenotypes, thereby creating substrate for natural selection to act upon. We use a system of two contrasting morphs of Arctic charr and as a first step we surveyed miRNA expression at four developmental stages thereby homing in on the miRNA genes that may have a bearing on the morphological and functional differences between the morphs. Differences in expression levels were detected in 72 miRNAs. Interestingly, the majority of these miRNAs (53/72) are evolutionarily stable and have been previously described as part of important developmental processes such as neurogenesis, erythropoiesis, skeleto- and myogenesis, specifically in craniofacial elements. Some miRNAs (e.g. the let-7 and miR430 families) show indications of differences in timing of expression. Other miRNAs (sal-miR-152 and sal-miR-451) exhibit sequence divergence. We are currently working on follow up experiments e.g. looking for the putative targets of the interesting miRNA candidates found in this study and defining their expression pattern using *in situ* hybridization in embryos derived from additional morphs and populations.

## Supporting Information

Figure S1 **Length distribution of reads in the small-RNA-seq data for all samples combined.** A major peak is observed at 22 nt, corresponding to the typical miRNA size.
(TIF)

Figure S2 **Length distribution of reads in individual miRNA-seq samples.** A–D: Small benthic (SB) stages 1–4; E–H: Aquaculture (AC) stages 1–4. Left panel: redundant reads, Right panel: unique reads.
(PPTX)

Table S1 **Background information and primers used for amplification of selected miRNA clusters.** The names of *S. salar* contigs and miRNAs in clusters used for primer design, sequence identity (%) between Arctic charr and Salmon, and forward and reverse primer sequences are shown.
(XLS)

Table S2 **Conserved Arctic charr miRNAs.** 326 known miRNAs were identified in the small-RNA-seq data. Included are miRNA names, miRNA sequences, number of raw reads per sample and names of miRNA orthologs.
(XLS)

Table S3 **Putative novel arctic charr miRNAs.** 427 novel miRNA candidates. Included are novel miRNA candidate IDs, miRDeep2 score (the log-odds score assigned to the hairpin by miRDeep2), significant (p<0.005) randfold p-value of the putative miRNA to form a hairpin structure, consensus mature, consensus star, consensus precursor sequences and genomic coordinates in Salmon.
(XLS)

Table S4 **Background data for** **figures 3** **and** **4** **.** Normalized number of reads in all 8 samples for miRNAs that showed differential expression between morphs and/or developmental time-points.
(CSV)

File S1 **R code used for differential expression analysis of miRNA-seq data.**
(R) Click here for additional data file.

File S2 **R code used to calculate relative expression (fold change) for each miRNA compared to stage 1 in AC.**
(R) Click here for additional data file.
