# Supplementary figures and images for: Patterns of MiRNA Expression in Arctic Charr Development
Source: PLoS One. 2014 Aug 29;9(8):e106084. doi: 10.1371/journal.pone.0106084 (PMC4149506; doi:10.1371/journal.pone.0106084)

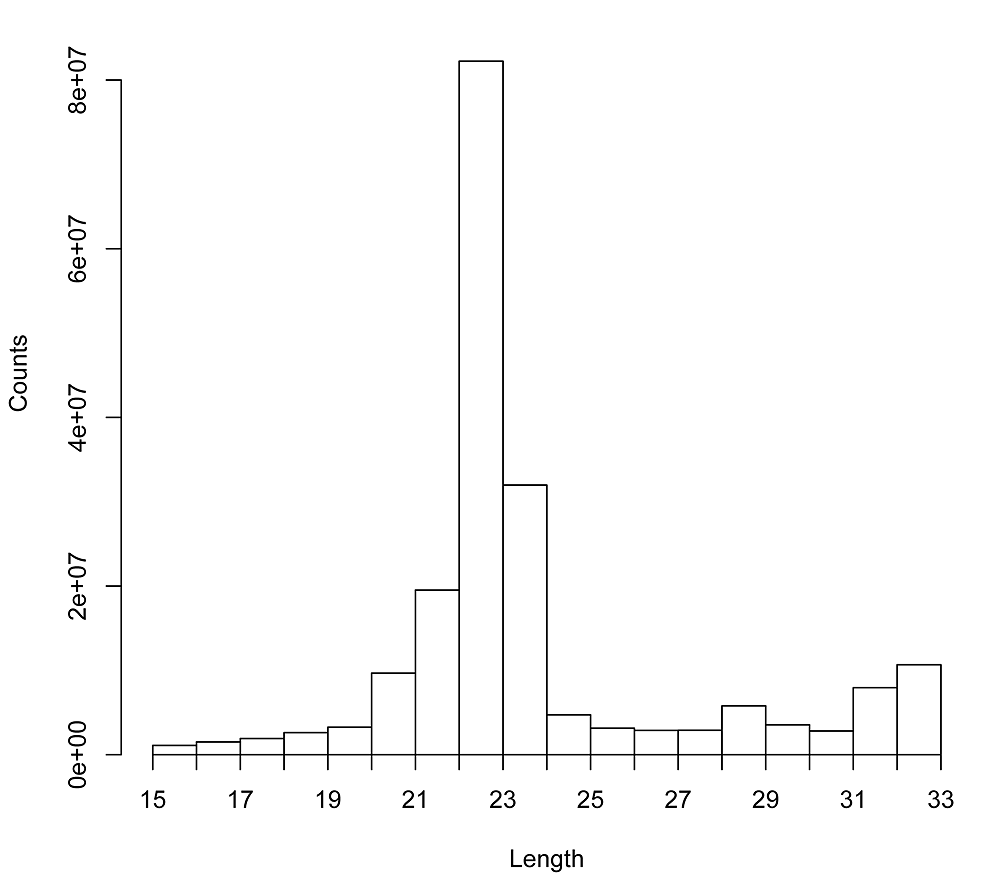

Supplement: Figure S1 — Length distribution of reads in the small-RNA-seq data for all samples combined. A major peak is observed at 22 nt, corresponding to the typical miRNA size. (TIF) [file pone.0106084.s001.tif]

## Slide 1
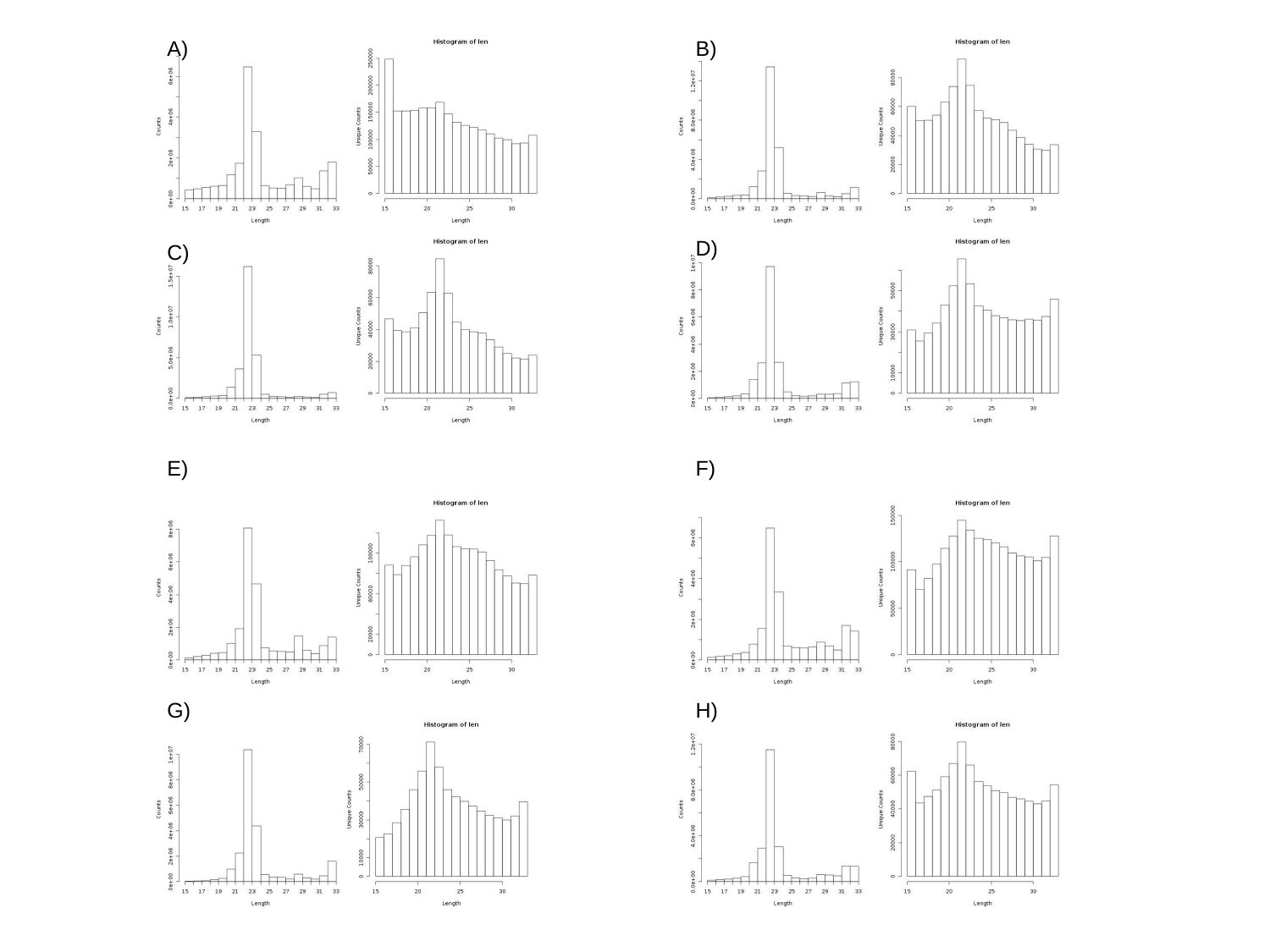

A)
B)
D)
C)
E)
F)
G)
H)

Supplement: Figure S2 — Length distribution of reads in individual miRNA-seq samples. A–D: Small benthic (SB) stages 1–4; E–H: Aquaculture (AC) stages 1–4. Left panel: redundant reads, Right panel: unique reads. (PPTX) [file pone.0106084.s002.pptx]
